# 

**Published:** 1896-06

**Authors:** 


					﻿Vol. XVI.
JUNE, 1896.
No. 6?
THE
HOMEOPATHIC pHY£lClA[\I,
A Monthly Journal of Homoeopathic Materia Medica
and Clinical Medicine.
"He u a freeman whom truth makes free, and all are slaves beside."
"Seek the Truth: come whence it may, cost what it will."
EDITED AND PUBLISHED BY
WALTER KI. JAMES, M. D.
PHILADELPHIA:
TTo. 1231 LOCUST STREET.
LONDON ;•
ALFRED HEATH & CO., 8ole Agents for Great Britain,
114 Ebury Street, Eaton Square.
early Subscription, $2.SO, invariably in advance. Single numbers, 2S ets.
Entered at the Philadelphia Post-Office as Second-Class Mall Matter.
				

